# A large asymptomatic Thornwaldt’s cyst incidentally discovered in a patient with chronic kidney disease: a case report and review of clinical implications

**DOI:** 10.1097/MS9.0000000000003757

**Published:** 2025-09-01

**Authors:** Ghazal Iftakhar, Muhammad Usama Bin Shabbir, Aiman Waheed, Naeema Natasha, Muhammad Hamza Gul, Abdul Baseer Wardak

**Affiliations:** a Department of Otolaryngology, Pakistan Institute of Medical Sciences (PIMS), Islamabad, Pakistan; b Internal Medicine Department, Pakistan Institute of Medical Sciences (PIMS), Islamabad, Pakistan; c Internal Medicine Department, Rawalpindi Medical University and Allied Hospitals, Rawalpindi, Pakistan; d Internal Medicine Department, Hayatabad Medical Complex, Peshawar, Pakistan; e Internal Medicine department, Razia Bahlol Hospital, Kabul, Afghanistan

**Keywords:** case report, chronic kidney disease, MRI, nasopharyngeal cyst, otorhinolaryngology, Thornwaldt’s cyst

## Abstract

**Introduction and importance:**

Thornwaldt’s cysts are benign nasopharyngeal anomalies that are often asymptomatic but can present diagnostic challenges and potential complications, necessitating clinical vigilance. Although these cysts are typically benign, their incidental discovery requires careful evaluation, particularly in asymptomatic patients with co-morbid conditions such as chronic kidney disease (CKD). This case underscores the importance of comprehensive imaging and ongoing monitoring for proper management of such nasopharyngeal anomalies.

**Case presentation:**

We present the case of a 28-year-old man with CKD who was incidentally diagnosed with a Thornwaldt’s cyst while being evaluated for recurrent epistaxis and dizziness. Initial clinical examination revealed nasal turbinate hypertrophy but with normal nasal patency. Magnetic resonance imaging of the brain and nasopharynx revealed a clearly defined, small cystic mass (1.1 × 1.2 cm) located on the posterior nasopharyngeal wall.

**Clinical discussion:**

Thornwaldt’s cysts generally have a favorable prognosis with conservative management. However, periodic imaging and clinical evaluation are essential to detect any symptomatic progression or complications that may require surgical intervention. Although the cyst was relatively large in comparison to typical literature reports, its benign nature, along with the patient’s lack of symptoms, allowed for conservative management. Given the patient’s underlying CKD, close monitoring was recommended to detect any changes in size or potential complications, including the development of obstructive symptoms or infection.

**Conclusion:**

Diligent monitoring through routine imaging is crucial for detecting and managing incidental findings such as Thornwaldt’s cysts, especially in patients with underlying health conditions like CKD. Routine follow-ups are necessary to ensure early intervention if symptoms develop or if the cyst changes in size, ensuring timely management of any potential complications.

## Introduction

Thornwaldt’s cysts are benign anomalies that are seen in the nasopharynx. They are sometimes referred to as Thornwaldt’s cysts or nasopharyngeal cysts. They are often discovered unexpectedly during routine imaging studies. These cysts originate from remnants of the notochord and are usually symptomless. However, they might occasionally produce general symptoms, making diagnosis difficult and needing medical expertise. The reported incidence of Thornwaldt’s cysts ranges from 1% to 4% in imaging studies^[1]^. Most are discovered incidentally during imaging for unrelated reasons^[2]^. Patients with chronic kidney disease (CKD) often undergo frequent imaging for systemic symptoms or complications, which can lead to the incidental detection of unrelated pathologies such as nasopharyngeal cysts. This highlights the importance of interpreting incidental findings within the clinical context, as patients with CKD may present with overlapping nonspecific symptoms such as dizziness or nasal bleeding. Symptomatic cysts may cause halitosis, foul-smelling discharge, or ear infections due to Eustachian tube obstruction^[3]^. Treatment is usually unnecessary unless it results in nasal obstruction, postnasal drip, or recurring infections due to size or location. Radiological interventions like computed tomography (CT) and magnetic resonance imaging (MRI) scans are crucial for diagnosis. On CT scans, Thornwaldt’s cysts appear as well-defined, round lesions deep within the nasopharyngeal mucosa, typically measuring 2–10 mm in diameter with low-density centers and no post-contrast enhancement^[4,5]^. High signal intensity, a thin wall, and no increase after contrast are observed on T2-weighted MRI scans^[6,7]^. Asymptomatic cysts generally require no intervention but may necessitate periodic imaging to monitor for any changes in size or characteristics^[8]^. Surgical techniques such as trans-nasal de-roofing or marsupialization are preferred for symptomatic individuals. This treatment involves making a minor incision in the cyst wall to drain fluid, ease symptoms, and lower the chance of recurrence^[9,10]^. This case study highlights the need to diagnose Thornwaldt’s cysts accurately to prevent unnecessary treatments and repercussions.

**HIGHLIGHTS**

- Thornwaldt’s cysts are benign anomalies that are seen in the nasopharynx.
- On CT scans, they appear as well-defined, round lesions deep within the nasopharyngeal mucosa, typically measuring 2–10 mm in diameter with low-density centers and no post-contrast enhancement.
- High signal intensity, a thin wall, and no increase after contrast are observed on T2-weighted MRI scans.
- They present intricate diagnostic challenges and potential complications, necessitating careful consideration.
- This case demonstrates the unexpected discovery of a Thornwaldt’s cyst in a 28-year-old man with chronic kidney disease.

## Methodology

This case report has been formulated based on the SCARE guideline checklist^[11]^.

### Case presentation

A 28-year-old male patient with a significant medical history of CKD, currently undergoing dialysis twice a week, presented to the outpatient clinic with a range of symptoms. Over the past month, he had experienced dizziness, imbalance, and recurrent nosebleeds, the latter of which required nasal packing. A nasal examination revealed congested mucosa and hypertrophy of the right middle and left inferior turbinates. Bilateral nasal patency was adequate, though a full endoscopic examination was not performed at this stage.

Given the patient’s medical history and the progression of his symptoms, laboratory investigations were ordered to assess for potential infections and to gain further insights into the underlying causes of his symptoms. The results of the hematological tests are summarized in Table 1, and the CKD workup is summarized in Table 2.

**Table 1:** Summary of blood workup and coagulation profile of the patient

Test Name	Result	Normal range
WBC (white blood cell count)	14 720/µL	4000–10 000/µL
RBC (red blood cell count)	3.73 million/µL	4.5–5.5 million/µL
H.B. (hemoglobin)	7.7 g/dL	13-–17 g/dL
Hematocrit	24.7%	40–50%
Mean corpuscular volume	66.1 fl	80–98 fl
Mean corpuscular hemoglobin	20.6 pg	27–32 pg
Mean corpuscular hemoglobin concentration	31.2 g/dL	31.5–34.5 g/dL
Platelet count	238 000/µL	150 000–400 000/µL
Neutrophils	84.4%	45–70%
Lymphocytes	10.2%	20–40%
Eosinophils	0.7%	1–6%
Monocytes	4.6%	2–10%
Basophils	0.1%	0.5–1%
PT (prothrombin time)	13.07 seconds	11–13 seconds
International normalized ratio	1.01	0.8-1.2
aPTT (activated partial thromboplastin time)	34.9 seconds	30–40 seconds

**Table 2:** CKD workup of the patient

Test name	Result	Normal range
Serum creatinine	3.9 mg/dL	0.6–1.2 mg/dL
Blood urea nitrogen	35 mg/dL	7–20 mg/dL
Estimated glomerular filtration rate	20 mL/min/1.73 m^2^	>90 mL/min/1.73 m^2^
Serum potassium	5.2 mmol/L	3.5–5.0 mmol/L
Serum calcium	8.9 mg/dL	8.5–10.5 mg/dL
Phosphate level	4.7 mg/dL	2.5–4.5 mg/dL
Urine protein/Creatinine ratio	0.5 g/g	<0.2 g/g
Urinalysis (Appearance)	Clear	Clear

### Hematological results interpretation

The patient’s **blood work** indicated the following significant abnormalities:

- **Severe anemia**: The **hemoglobin** level was found to be **7.7 g/dL**, well below the normal range of **13–17 g/dL**. This could be attributed to his **CKD**, where anemia is a common complication due to decreased erythropoietin production.
- **Elevated white blood cell (WBC) count**: His **WBC** count was **14 720/µL**, significantly higher than the normal range of **4000–10 000/µL**, indicating a possible underlying infection or inflammation. The increase in **neutrophils (84.4%)** may suggest a bacterial infection or an inflammatory response.
- **Microcytic anemia**: The **mean corpuscular volume of 66.1 fl** is indicative of **microcytic anemia**, which is commonly associated with iron deficiency, though it may also be seen in CKD-related anemia.

**Figure 1.**
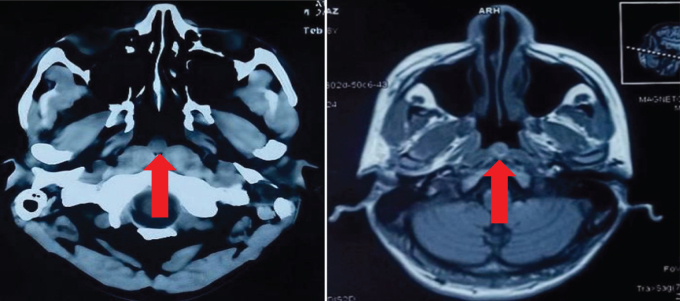
T2-weighted fat-suppressed fluid-attenuated inversion recovery image transverse views of magnetic resonance scans, showing hyperintense rounded cystic area representing Thornwaldt’s cyst. It measures 1.1 × 1.2 cm (anteroposterior × transverse diameter).

**Figure 2.**
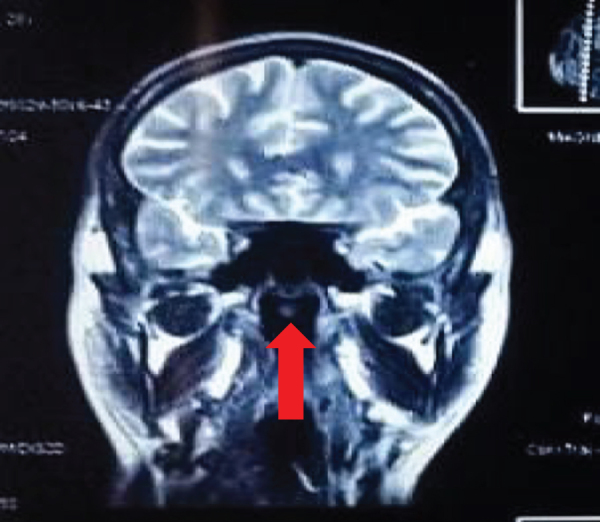
T2-weighted fat-suppressed fluid-attenuated inversion recovery image – coronal view of magnetic resonance scans, showing hyperintense rounded cystic area representing Thornwaldt’s cyst.

These abnormalities warrant close monitoring, as they may influence clinical decision-making, particularly in managing any potential infections, inflammatory responses, or anemia, all of which can complicate his current health condition. The cerebrum and cerebellum showed normal cortical sulcation and signal intensity on MRI, with the interhemispheric fissure near the midline. No signs of elevated intracranial pressure were found. Cerebral ventricles appeared normal. All other brain structures were intact, with no abnormalities. Furthermore, the pituitary and sella glands were normal, and the parasellar area lacked distinguishing traits. The cerebellopontine angle and internal acoustic meatus were also within normal ranges. A noticeable cystic area measuring 1.1 × 1.2 cm was located in the back wall of the nasopharynx. High signal intensity, a thin wall, and no increase after contrast are observed on T2-weighted MRI scans. The imaging findings show similar intensity on T1-weighted images and increased intensity on T2-weighted and FLAIR images. Figure 1 shows the cyst in T2-weighted fat-suppressed fluid-attenuated inversion recovery image transverse views of magnetic resonance scans. Figure 2 shows the cyst in T2-weighted fat-suppressed fluid-attenuated inversion recovery image – coronal view of magnetic resonance scan.

### Clinical significance of the cyst’s size

The size of the **Thornwaldt’s cyst** in this case, measuring **1.1 × 1.2 cm, is far larger** than the typical **2–10 mm** range often reported in the literature. While most Thornwaldt’s cysts are small, asymptomatic, and benign, the **unusually large size** of this cyst raises concerns regarding its potential for **compressive symptoms** or future complications, despite its current lack of symptoms. This emphasizes the need for close monitoring, particularly in the context of **CKD**, where immunosuppression from dialysis or underlying conditions may increase the risk of infection or growth of such cysts.

### Differential diagnoses

The nasopharyngeal cyst detected on imaging should be differentiated from other possible nasopharyngeal lesions, including

- **Choanal polyps**: These are benign growths that could present with similar symptoms, including nasal obstruction or bleeding.
- **Adenoid cystic carcinoma**: Although rare, this malignant tumor can present with nasopharyngeal masses and symptoms such as **nosebleeds** and **nasal obstruction**, requiring early identification and differentiation.

In this case, there is no indication of malignancy, but ongoing monitoring is advised to ensure no future complications arise from this **benign cyst**.

### Management

The patient was asymptomatic with no signs of nasopharyngeal obstruction or infection, so no immediate intervention was required. He was advised to manage mild symptoms with steam inhalation. The Thornwald’s cyst, a benign anomaly, may resolve on its own. However, should it increase in size or cause symptoms, surgical options like excision or marsupialization could be considered. The increased WBC count suggested an underlying infection. Empiric antibiotics were initiated targeting common nasal and sinus pathogens, and the patient was monitored for any signs of worsening infection. Follow-up blood work was scheduled to ensure resolution of the infection. Given the patient’s CKD, which may impair immune response, the cyst’s size and the patient’s overall condition will be monitored closely. A follow-up appointment was set in 6 months to reassess the cyst and check for any emerging symptoms.

## Discussion

Thornwaldt’s cysts are exceedingly rare benign lesions found within the nasopharynx that usually do not cause symptoms and are often discovered incidentally during diagnostic imaging^[11,12]^. Thornwaldt’s cysts, although benign, can pose diagnostic challenges and require extensive evaluation^[13]^. A 28-year-old individual with CKD experienced nosebleeds, dizziness, and slurred speech, prompting an investigation into nasopharyngeal abnormalities. Enlarged nasal turbinates and congested nasal mucosa observed during the initial examination suggested a nasopharyngeal lesion^[14]^. On MRI, Thornwaldt’s cysts appear with smooth-bordered cystic lesions, showing similar signal intensities on T1-weighted images and brighter than normal on T2-weighted and FLAIR images^[15,16]^. These imaging features are essential for distinguishing Thornwaldt’s cysts from other nasopharyngeal conditions, such as choanal polyps or adenoid cystic carcinoma^[17]^. The comparative analysis of imaging in nasopharyngeal lesions highlights the distinction between Thornwaldt’s cysts and other pathologies like adenoid cystic carcinoma, which presents with more irregular borders, and choanal polyps, which typically show homogeneous enhancement on imaging. The MRI findings of Thornwaldt’s cysts are crucial in ruling out these malignancies, providing reassurance of the benign nature of the cyst in this patient. Notably, this patient’s cyst was larger than those typically reported in the literature, which usually describes Thornwaldt’s cysts as small lesions. The size of the cyst in this case added complexity to the clinical decision-making process, but the benign nature of the lesion was confirmed through imaging, ruling out malignancy.

The asymptomatic Thornwaldt’s cysts were managed with reassurance and symptom relief. The patient was informed of the cyst’s benign nature and the potential for spontaneous regression^[18]^. Current guidelines support this conservative approach, emphasizing avoiding invasive procedures unless symptomatic progression necessitates intervention. Long-term follow-up is necessary to monitor any changes in cyst size or the development of symptoms^[19]^. Thornwald’s cysts generally have a favorable prognosis with conservative management^[20]^. However, periodic imaging and clinical evaluation are essential to detect symptomatic progression or complications that may require surgical intervention. Given the patient’s CKD, which could increase vulnerability to infection or other complications, close follow-up was advised, even in the absence of symptoms.

Histologically, Thornwaldt’s cysts typically show a thick fibrous wall and mucous glands, confirming their benign nature. While controversy exists regarding the management of larger asymptomatic cysts, particularly in terms of surgical intervention, literature generally supports conservative management for cysts without significant symptoms. Larger cysts, like the one in this case, are rare but are still unlikely to develop malignancy. The risk of malignancy in Thornwaldt’s cysts is extremely low, and imaging and histopathology help rule out more concerning diagnoses. The decision to recommend follow-up was driven by the unusual size of the cyst, even though no malignancy was suspected. Monitoring is essential to ensure that no symptomatic progression or complications, such as infection or obstruction, arise, which could necessitate surgical intervention^[8,9,12,19]^.

The significance of comprehensive imaging in diagnosing and managing nasopharyngeal anomalies, including Thornwaldt’s cysts, cannot be overstated, especially in asymptomatic patients with underlying chronic conditions such as CKD. Early identification through meticulous radiological assessment allows for timely intervention if symptomatic progression occurs, emphasizing the critical role of incidental findings on routine imaging in improving patient care and outcomes. While Thornwaldt’s cysts are typically benign and asymptomatic, their incidental discovery in patients undergoing diagnostic evaluation underscores the need for thorough clinical assessment and radiological imaging to ensure accurate diagnosis and appropriate management^[21]^. Further research and long-term studies are necessary to understand better the natural progression and the best management approaches for rare nasopharyngeal growth.

## Conclusion

In conclusion, diligent and vigilant monitoring during routine imaging is crucial for detecting and managing unexpected findings like Thornwaldt’s cysts, particularly in patients with underlying health conditions. Given their often asymptomatic nature, these cysts can pose diagnostic challenges and may necessitate ongoing observation to preempt potential symptoms or complications. Routine examinations and follow-ups are critical for ensuring early care in the event of progression or complications and monitoring any changes in cyst size or accompanying symptoms. This preventive technique is critical for detecting cyst development and accompanying problems such as recurring infections or nasopharyngeal blockage.

### Patient’s perspective

The patient was reassured upon learning that Thornwaldt’s cyst is a benign and generally asymptomatic condition. Since he was not experiencing any symptoms at the time of diagnosis, no immediate intervention was required, and steam inhalation was suggested for symptom relief. However, due to an identified infection reflected by an elevated WBC count, the patient was treated with empiric antibiotics targeting common nasal and sinus pathogens. This was done to address any potential underlying infection that could be contributing to the symptoms. The patient was informed that, in most cases, Thornwaldt’s cysts are benign and may resolve on their own over time. Given the cyst’s larger-than-expected size compared to typical reports in the literature, the patient was educated on the importance of monitoring its progression. He was advised that, should symptoms or complications arise in the future, surgical options like excision or marsupialization could be considered. A follow-up examination was scheduled for 6 months later to assess any changes in the size of the cyst and to monitor for the emergence of any new symptoms. This follow-up was particularly important given the cyst’s unusual size and the patient’s CKD, which may predispose him to complications. The patient understood the need for ongoing surveillance to ensure that no further complications, such as obstruction or infection, occurred.

## Data Availability

The authors declared the data availability statement.
